# A Multianalytical Investigation to Preserve Wall Paintings: A Case Study in a Hypogeum Environment

**DOI:** 10.3390/ma16041380

**Published:** 2023-02-07

**Authors:** Maria Antonietta Zicarelli, Mauro Francesco La Russa, Maria Francesca Alberghina, Salvatore Schiavone, Raffaella Greca, Paola Pogliani, Michela Ricca, Silvestro Antonio Ruffolo

**Affiliations:** 1 Department of Biology, Ecology and Earth Sciences (DiBEST), University of Calabria, Arcavacata di Rende, 87036 Cosenza, Italy; 2 S.T.Art-Test, 93015 Niscemi, Caltanissetta, Italy; 3 Department of Economics and Management, University of Tuscia, Largo dell’Università, 01100 Viterbo, Italy

**Keywords:** the Sotterra church, wall painting, conservation, restoration, nondestructive analysis, microclimate, efflorescence

## Abstract

In the present study, a diagnostic approach was used to analyze the wall painting in the apse of the Sotterra church at Paola, in the province of Cosenza, Italy. The Sotterra church is nowadays located 6 m under the ground level. The presbytery area houses valuable pictorial evidence attributable to different phases. The oldest painting, visible in the apse area, dates back to the 11th–12th centuries, and it represents the subject of the present study, while the later decorations are placed in a chronological range from the 14th to the 15th centuries. Due to the peculiar environmental conditions, the conservation of subterranean sites represents a debated issue and must be properly investigated. For this reason, in this research, noninvasive analysis and laboratory-based methods were planned to obtain information about both the composition of original materials and the mechanisms and causes of alterations affecting the wall painting in the apse. Simultaneously, an environmental monitoring campaign of the indoor climate for the duration of nine months was conducted. The results highlighted the use of natural mineral pigments such as ochres and earths. The analysis of the painting stratigraphy revealed that the mural painting consists of two plaster layers characterized by lime-based binder. Moreover, the presence of a high amount of calcium sulfate has been discovered; this latter result, combined with the monitoring of the microclimate, allowed for the establishment of the crystallization and the condensation risks which occur on the investigated surfaces.

## 1. Introduction and Historical Background

The Sotterra church is located in Gaudimare quarter, 3 kilometres from Paola (Cosenza, Italy). Its foundation and architectural phases still pose several questions to art historians. The founding date of the church is hotly debated by scholars: some opine it may have been founded between the 7th and 8th centuries [1,2,3,4]; others suggest it was around the 9th century [5]; others propose the 12th century [6]. According to the most sustained historical hypothesis, the church was built between the 9th and 10th centuries, during the Byzantine dominion over the whole region. Most likely, starting from the 12th century, the building underwent significant architectural changes following the adaptation to the Latin cult, related to the presence in the area first of Benedictine monks and then to the presence of monks of the Order of S. Giovanni in Fiore [5]. From the 16th century, the site fell into oblivion, and it was only in 1876 that it was brought to light during the construction of the upper Madonna del Carmine Church [7].

As suggested by its name, the Sotterra church nowadays appears as an underground site. Despite some historians’ statements—such as those of Isnardi [1] and Frangipane [2], which define the site as a hypogeum or a crypt—the building was constructed at ground level [5]. Its burial was caused by several landslides that occurred at different times, as demonstrated by the evidence discovered from late 20thcentury archeological excavations.

The architecture of the site, in its current appearance, reveals a rectangular single nave, with two lateral arcosolia, surmounted by three round arches, ending with an apse [5]. The presbytery area contains various mural paintings attributable to different chronological phases. Paintings above the lateral altar represent: on the left the Virgin “Galaktotrophousa” nursing the child, holding the pomegranate; and on the right, a saint monk identified as Sant’Antonio Abate, marked on his sides with the letter ‘S’ and with the syllables ‘IUS’. Some sources [5], on the basis of stylistic comparisons, ascribe both to the 15th century.

A second decorative phase, on the side walls of the apse, shows the theme of the Annunciation, carried out in two frames. On the left, the angel is painted with a light drapery decorated with Latin crosses, with wide wings, facing the Virgin, who appears instead with a sober robe and holding a book. This last pictorial representation is datable between the 14th and 15th centuries [5], and it was studied from a scientific viewpoint in previous research [8].

**Figure 1:**
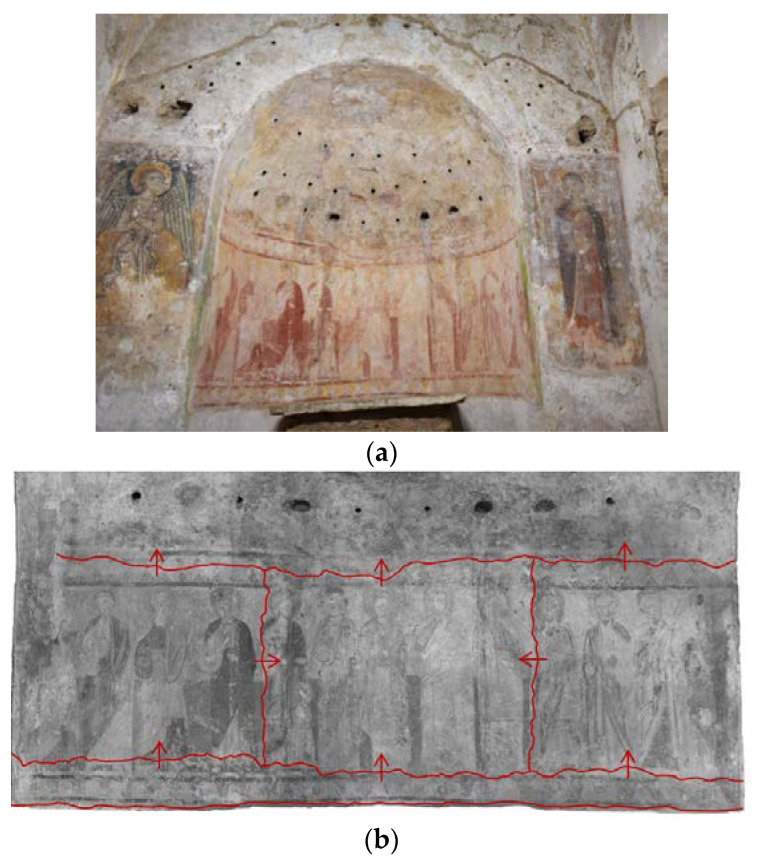
(**a**) Wall painting in the apse of the Sotterra church; (**b**) mapping (in red) of the overlapping pattern of the *giornate*.

While these two paintings relate to the last phase of the building, the paintings in the apse are much older. The latter represent the subject of this study (Figure 1a). The extremely incomplete state of the painting makes it difficult to judge a stylistic and iconographic interpretation. To date, the proposal put forward by art historians, which identified the figures as male and female saints, has been replaced by the hypothesis according to which the iconographic theme represented would be the Ascension, contaminated by the theme of the *Maiestas domini*. In the apsidal basin the Virgin is represented in the center of the scene, beside the Twelve Apostles, while in the semidome Christ is depicted, seated on a throne, in the almond, no longer observable due to its poor state of conservation.

Thanks to a visual observation conducted in raking light, the presence of several *giornate* was detected (Figure 1b). The *fresco* painting technique implies that the mortar during carbonation process incorporates pigments. For this reason, the *intonaco* must always be applied gradually, as the painting is carried out, so that it is still wet when it is painted [5]. By observing the overlapping correlations, it was possible to determine that the painting was made by at least five *giornate* (portions of painting that had been made in a day): the first *giornata* is applied on the semi-dome; the second *giornata* includes the five central figures; the third and fourth *giornate* overlap the second laterally; the last *giornata* includes the lower decorative frame. The division of the space is regulated by several snapped lines obtained by stretching a string, pulling it back, and releasing it so that it leaves a mark [6]. It can be determined that the center of the apse was made vertically; the other outlines the background horizontally.

A diagnostic study has been carried out on this painting by means of noninvasive analysis (microclimatic monitoring, portable X-ray fluorescence, infrared imaging, and UV-induced visible fluorescence imaging) and laboratory-based methods (ionic chromatography, optical, and electron microscopy). The aim was to obtain information about the composition of original materials, as well as about the mechanisms of alterations affecting the wall painting.

## 2. Materials and Methods

In ancient times, the construction of hypogea was very common. These could be built entirely by man or could have a natural origin (e.g., caves) and mainly present functions related to cult (e.g., mithraea and hermitages) and burial (e.g., catacombs and necropolises) [8,9,10]. However, the Sotterra church was not initially built as a hypogeum but has become an underground site due to natural events. Underground sites are generally characterized by high relative humidity and constant temperatures that show only slight seasonal variations [11].

Humidity represents the first cause of alteration to the mural paintings [9]. The porous structure of the plaster is able to convey the water and the salts dissolved in it. If this equilibrium is perturbed by external factors such as the influx of visitors, the penetration of air currents, and the presence of lighting sources, alteration phenomena like saline efflorescence, growing of biofilms, and recrystallization can occur.

To determine a conservation plan, it is advisable to define both the composition of original materials and the mechanisms and causes of alterations affecting the monument. A diagnostic campaign must be conducted, which has the purpose not only of the surface’s investigation, but also of the environmental characterization. The diagnostics concerned are:

- The investigation of the forms of alteration and degradation on the surfaces of the wall painting through both noninvasive investigations in situ and invasive investigations conducted in the laboratory;
- The investigation of constitutive materials through both noninvasive investigations in situ and invasive investigations conducted in the laboratory;
- Environmental microclimatic monitoring with detection of temperature (T) and relative humidity (RH);

### 2.1. Microclimate Monitoring

**Figure 2:**
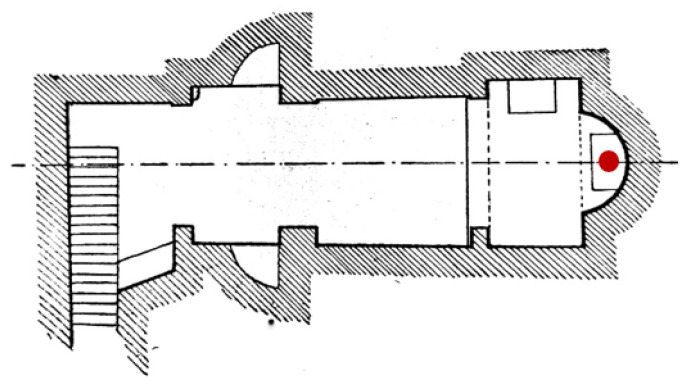
Position of the datalogger in the presbytery area of the Sotterra church.

The Data Logger EL-USB-LCD from EasyLog has been used, which measures from −35 to +80 °C(−31 to +176 °F) and from 0 to 100% humidity (RH) range. It was positioned on the upper surface of the main altar, in correspondence of the apse in order to obtain information about temperature (T) and relative humidity (RH %) (Figure 2). The acquisition interval was set as 15 min.

Monitoring was performed for a total of about nine months, from December 2021 to September 2022. Data were then processed to determinate the daily, monthly, and seasonal average, minimum, and maximum of both temperature and relative humidity. Furthermore, the parameters obtained were compared with the outdoor climatic data provided by ARPACAL (Agenzia Regionale per la Protezione dell’Ambiente della Calabria).

### 2.2. In-Field Methods

Infrared Imaging (IR), Portable-X-Ray Fluorescence (PXRF), UV-Induced Visible Fluorescence Imaging (UVF Imaging), and Infrared Thermography were conducted using portable equipment.

Infrared imaging is a nondestructive analysis based on the emission of IR-radiation used to examine surfaces. The purpose of the investigation is to detect characteristics of the plaster that would not otherwise be visible, such as, for example, the presence of any underdrawings made from carbonaceous materials, *pentimenti* (earlier images, forms, or strokes that have been changed and painted over) made by the artist, and restoration interventions. A CCD photo sensor (MADATEC 28.2 MP multispectral system) with filters centered at 850 nm and 950 nm of the NIR spectrum and 1 s of exposure is used for the IR images grabs.

PXRF was performed to obtain a semiquantitative elemental composition of the painted layer. The portable XRF spectrometer used for the multielemental analysis of the pigments consists of the X-ray tube (Mini-X-Amptek) equipped with a rhodium (Rh) target and operating at a maximum working voltage of 40 kV and maximum current of 0.2 mA. The detection of the characteristic X-ray radiation emitted by the sample is as a function of the energy (Energy Dispersive: ED-XRF) and allowed by a Silicon Drift Detector system (X-123 SDD—Amptek) with 125–140 eV FWHM at 5.9 keV Mn K_ line Energy resolution (depending on peaking time and temperature), collimator 1 mm. The detection range of energy is from 1 keV to 40 keV, with a maximum rate of counts up to 5.6 x 105 cps.

The primary beam is positioned perpendicular to the sample, while the detector is positioned at 40 degrees with respect to the primary beam. A dedicated control software allows researchers to administrate measurements and acquisitions. For the analysis of the selected areas, the following measurement parameters are set in order to ensure a good spectral signal and to optimize the signal-to-noise ratio (SNR): voltage 35 kV; current 80 microA; acquisition time 40 s per area; working distance 1 cm.

**Figure 3:**
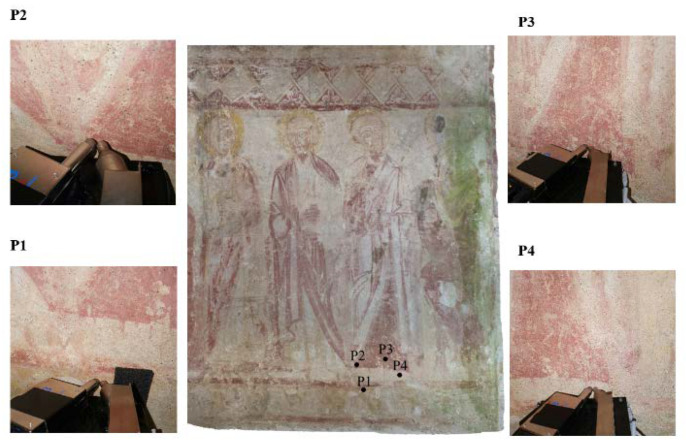
Measurement areas by PXRF of the apse wall painting.

PXRF was employed on the most representative pigmented areas, as shown in Table 1 and Figure 3.

UV-Induced Visible Fluorescence imaging is a nondestructive technique and allows the study of large surfaces without the need for sampling [12]. The phenomenon of UV fluorescence can be observed when some materials, suitably excited by a source of UV radiation, emit luminescence. It is a technique widely used to characterize and differentiate materials and substances on the surfaces, to understand the state of conservation and to reveal past treatments [13].

A CCD photo sensor (MADATEC 28.2 MP multispectral system, Madatec srl, 20060 Pessano con Bornago, Italy) is used for the UV-radiation stimulated optical fluorescence acquisitions, equipped with an UV-IR cut filter to allow the detector to only reach the visible fluorescence radiations emitted by the examined materials. Visible fluorescence is induced by ultraviolet light sources (two filtered LED sources with the emission peak centered at 365 nm, placed at 45 degrees with respect to the observed surface).

### 2.3. Laboratory-Based Methods

Eight samples were collected from the painting surface of the wall painting to conduct investigations like polarized optical microscopy (POM), ionic chromatography (IC) and scanning electron microscopy (SEM) coupled with energy-dispersive spectroscopy (EDS).

POM, with a Zeiss Axiolab microscope equipped with a digital camera, was conducted on thin sections to characterize mortar aggregate, to determine its conservation state, and to understand the stratigraphy of the pictorial layer.

All samples contain ionic species dispersed within the porous substrates. It follows that, with regard to the microclimatic conditions, cycles of dissolution and crystallization can potentially occur according to the different saline phases present in the substrate.

Therefore, it is essential to determine the nature of the salts dissolved in the substrate and to characterize the degradation forms that can be induced in the constituent material due to repeated crystallization cycles. For this reason, IC was performed to quantify cations (NH₄⁺, K⁺, Na⁺, Mg^2⁺^, and Ca^2⁺^) and anions (Cl^−^, NO_3_^−^, and SO_4_^2−^) on the superficial layer.

**Figure 4:**
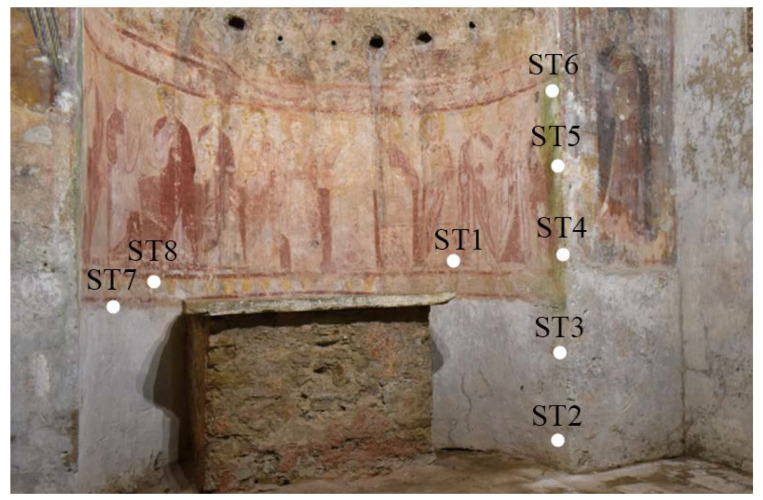
Sampling points for the apse mural painting.

SEM-EDS was employed to achieve information about the morphologies and chemical compositions of the binder and the pigments. For this purpose, Ultra-High-Resolution SEM (UHR-SEM)—ZEISS CrossBeam 350 equipment, as well as a spectrometer EDS—EDAX OCTANE Elite Plus—Silicon drift type, were used. Table 2 and Figure 4 briefly describe the sampling location and the diagnostic methods performed.

## 3. Results

### 3.1. Microclimate Monitoring

**Figure 5:**
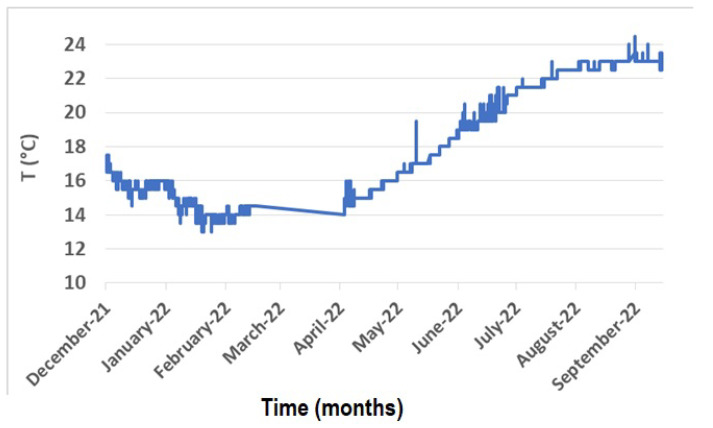
Temperature values measured inside the church for a period of nine months.

In the investigated period, the indoor temperature range has a minimum of 13 °C recorded in January and a maximum of 24 °C measured in September (Figure 5). More generally, we can state that the minimum temperature values are reached during January and February while the maximums are recorded during August and September.

The indoor temperatures show gradual variations during the monitoring, with monthly averages fluctuations ranging between 1 and 3 degrees from month to month. In addition, the indoor climate is characterized by minimal daily variation that goes from 0 to 1 degree and rarely from 0 to 1.5 degrees. Only in an exceptional case, on May 15th, the parameters of temperature show a significant increase of 2 degrees within 15 min, related to a massive frequentation of the site.

**Figure 6:**
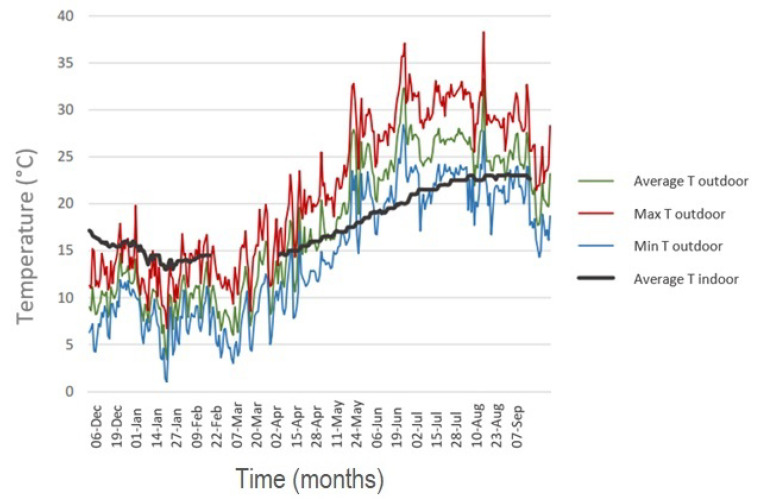
Temperature values measured in the church and the ones measured outside for a period of nine months.

The indoor microclimate is characterized by a high thermal stability, even considering the external daily variations of more than 10 degrees (Figure 6).

The minimum external temperatures are recorded from January to March, with a minimum of 0.9 °C measured in January. The maximum outdoor temperature values are recorded during summer, from June to August, with maximum temperature values exceeding 38°C. Due to the thermal inertia of the building, starting from May and during the summer, the indoor temperature values are lower than the external ones, while on the contrary, in winter the internal temperatures are higher than the ones acquired outdoors.

**Figure 7:**
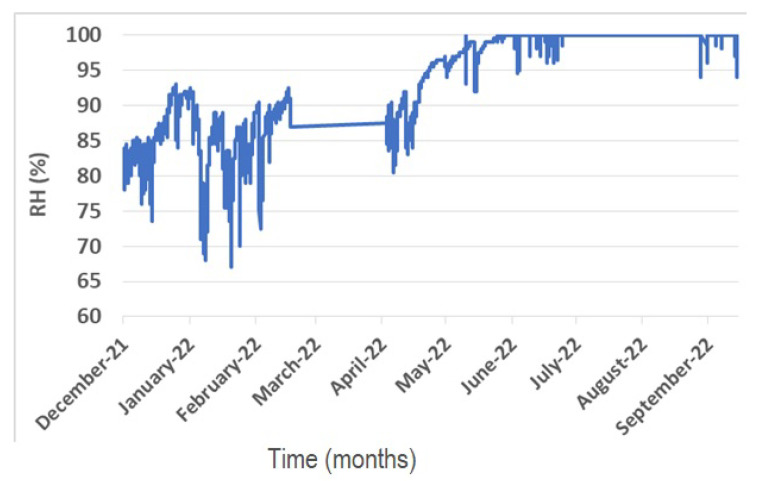
Relative humidity values measured inside the church for a period of nine months.

The microclimate of the Sotterra church is characterized by high relative humidity values, in keeping with the hypogea environments (Figure 7).

The average relative humidity recorded indoor during the monitoring is 92%. The lowest value is acquired during winter, in January, (67%,) while the condition of air saturation is reached in summer, starting from June. The results show wide daily oscillations of the relative humidity values during winter, which sometimes reach 12% variation. During spring, the relative humidity significantly increases, reaching 100% by June and maintaining the condition of air saturation until the first half of September.

**Figure 8:**
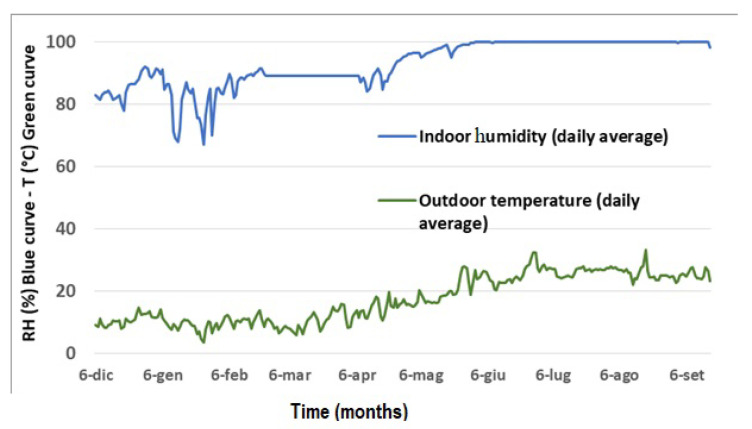
Daily average humidity (indoor) and daily average temperature (outdoor) for a period of nine months.

It is not possible to establish the relationship between the relative humidities recorded within the church and the external ones due to a lack of data relating to 2021 and 2022 in the ARPACAL archive. Nevertheless, a correlation between the indoor relative humidity and the outdoor temperature is evident (Figure 8). The trends of microclimatic data show that at the beginning of the summer period, the relative humidity inside the church increases as the outside temperature increases.

### 3.2. In-Field Methods

**Figure 9:**
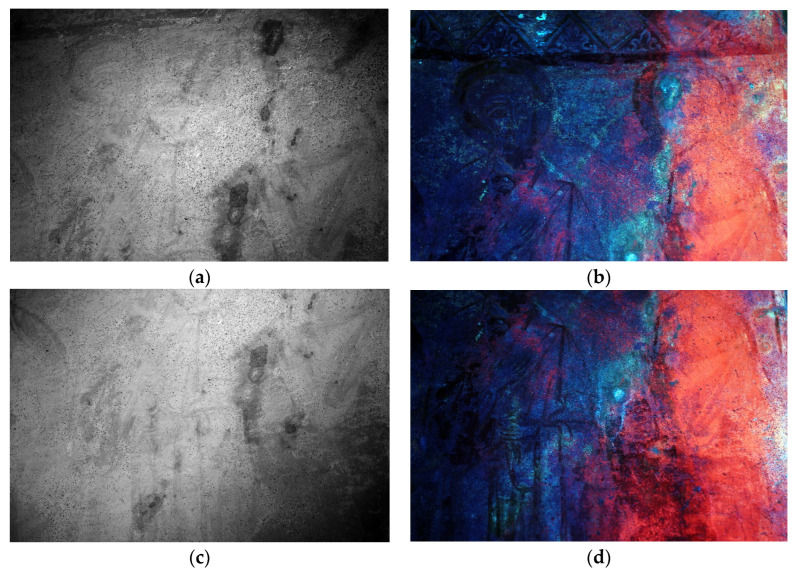
Infrared images (950 nm) (**a**,**b**); UV fluorescence images (**c**,**d**).

The IR images show no evidence of a preparatory drawing (Figure 9a,b) (dusting, incisions, or others) since the painting was created using ochres and it is difficult to observe under infrared radiation.

The thin pictorial layers applied directly on the plaster surface are perceptible due to the slight absorption at these wavelengths (950 nm). Greater absorption (darker area) is recorded in the areas affected by biological growth (see UV fluorescence).

Throughout the UV-Induced Visible Fluorescence imaging it was possible to observe the brilliant red luminescence emission on the left part of the wall painting (Figure 9c,d). This is related to the presence of biological growth that appears as a blue-green in the VIS image [13].

Biological growth is also observed in areas where it is not yet perceptible by visible light. In the central area of the apse, the whitish fluorescence coloring emphasizes the path of the water drains from the holes on the semidome.

This fluorescence might be attributed to the calcareous encrustations and to the efflorescence phenomena on the pictorial surface. In this case, it is also possible to observe some reddish fluorescence spots, attributable to microbiological species, which are characterized by lower florescence (such as the left part of the painting).

Four areas were selected to characterize the composition of the main colors on the wall surfaces.

**Figure 10:**
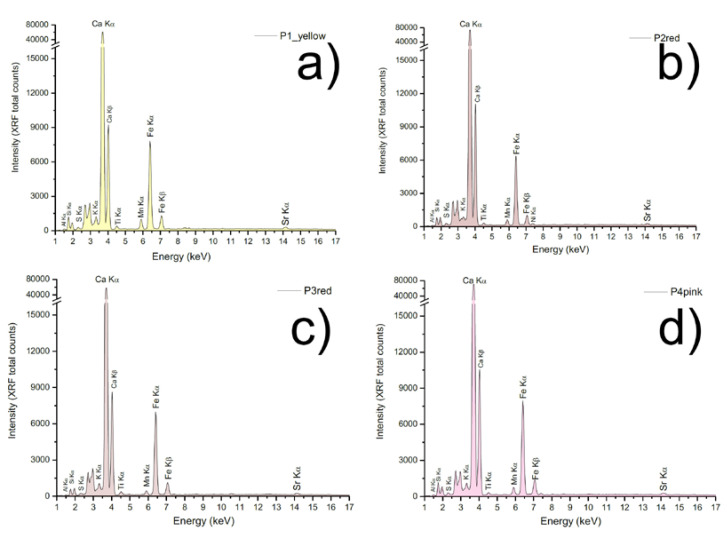
PXRF spectra acquired on the apse mural painting. P1, Yellow layer (**a**); P2, Red layer (**b**); P3, Red layer (**c**); P4, Pink layer (**d**).

The results suggest a restricted color palette, only composed of two main pigments (Table 3 and Figure 10). The signals of iron, manganese, silicon, aluminum, and potassium in all of the detected area suggest the presence of iron oxides, manganese dioxide, and clay silicates-based pigments. Even if the PXRF shows no significant elementary changes in pigments composition, the yellow tint is characteristic of iron hydroxides while the red one is related to iron oxides [9]. The pink pigment, used to paint the background, the Apostles’ robes, and the skin tones is most likely obtained by mixing the red pigment with some lime or calcium carbonate-based pigment [9]. This hypothesis is in part corroborated by the signal of calcium, which is also related to the plaster preparation.

### 3.3. Laboratory-Based Methods

The ionic species present in the solution inside of the plaster constituting the mural painting have been investigated. The main salts detected in the walls are carbonates, sulfates, chlorides, and nitrates.

The most abundant sulfates species found in walls are gypsum (CaSO_4_ 2H_2_O), magnesium sulfate (MgSO_4_ H_2_O), sodium sulfates like thenardite (Na_2_SO_4_), and mirabilite (Na_2_SO_4_ 10H_2_O) [14].

**Figure 11:**
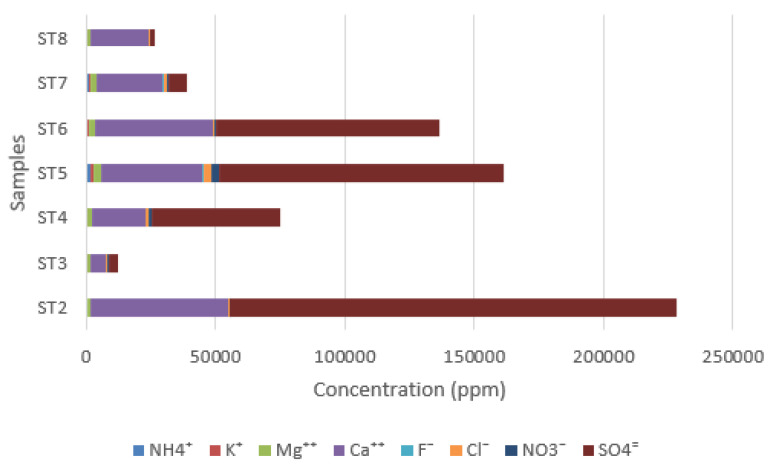
Ionic concentrations measured in the samples.

Figure 11 shows the different anionic and cationic concentrations (in ppm) obtained by ion chromatography (IC).

IC analysis shows no evidence of sodium ions, and considering the low contribution of magnesium ions, gypsum crystallization might occur. This could happen if the ambient relative humidity becomes lower than the equilibrium relative humidity (RH_eq_) of the saturated solution of the salt in the system [15]. Gypsum is characterized by the highest deliquescence RH, which is around 99.6% at 20 °C [15,16]. This means that the gypsum phase is stable even in extremely humid conditions.

**Figure 12:**
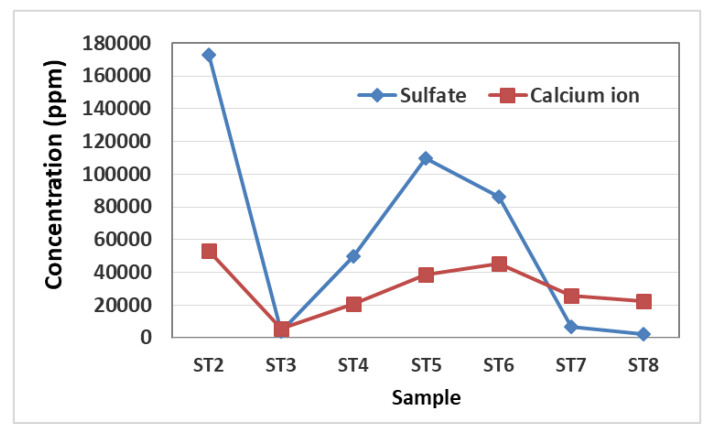
Concentrations trends of calcium and sulfate ions in the samples.

The strong correlation between the concentration of calcium ions and sulfate ions, expressed in Figure 12, corroborates the hypothesis of the presence of the gypsum phase.

**Figure 13:**
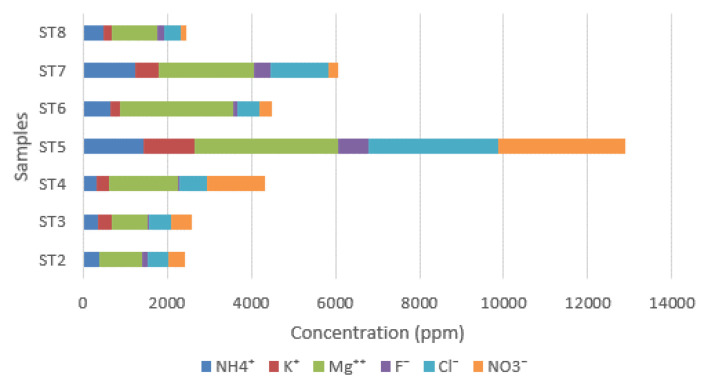
Ionic concentrations measured in the samples, calcium and sulfate ions excluded.

Sulfate ions could have several origins: they can come from the original materials or have different founts. In fact, they can be present as impurities in the plaster; however in the case of the apse mural painting of the Sotterra church, it is assumed that the calcium sulfate comes from the soil surrounding the building. Sulfate species are dissolved in soil water, and for capillary action may crystallize on the wall surfaces. In Figure 13, the ionic concentrations, excluding calcium and sulfates, have been reported.

Analyzing the contribution of other salt species determined by ion chromatography, and excluding calcium and sulfate concentrations, reference must also be made to the nitrates and chlorides present, albeit in minimal concentrations in the analyzed samples. Potassium and calcium nitrates generally come from the ground and are produced starting from the microbiological activity that decomposes organic nitrogenous products. The presence of chlorides is attributable to the proximity of the site to the coast, since the wind action promotes the transport of water particles coming from the sea.

The latter, in contact with the ground, are brought into solution with the other salts in the soil and, by capillarity, the chloride ions are conveyed within the porous structure of the wall.

**Figure 14:**
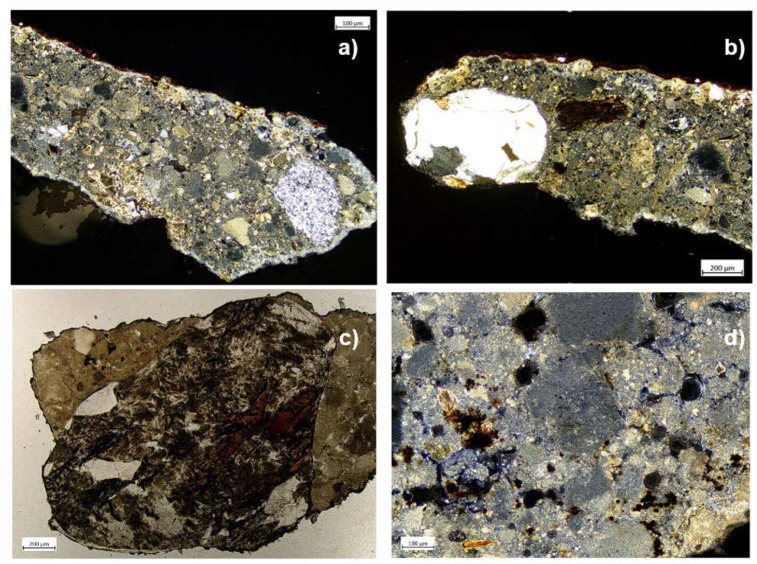
Images obtained by polarized optical microscopy. ST1 sample observed under crossed Nicols (**a**,**b**); ST8 sample observed under crossed Nicols (**c**); ST7 sample observed under parallel Nicols (**d**).

Thin sections were made for ST1, ST7, and ST8 samples. By POM analysis it is possible to achieve information about the execution techniques and the materials used in the mural painting’s realization (Figure 14).

In particular, an aggregate characterization was conducted. All samples are characterized by a poorly sorted aggregate, in which it is possible to recognize monocrystalline and polycrystalline granules. Grains present a dimensional range from a few microns up to a millimeter, a roundness ranging from angular to rounded, and a sphericity from high to low. The aggregate consists almost entirely of carbonate rock fragments, while quartz crystals, biotite, quartzite grains, and oxides are more rarely found in the samples observed. It is possible to highlight primary and secondary porosities, such as microcracks, linked to degradation phenomena. In addition, interparticle and intraparticle porosities were observed. Sample ST1 presents a thin pictorial film on the surface of the plaster whose color appears reddish to brown under crossed Nicols (XN) and shows that the pigmented layer has not penetrated the plaster.

**Figure 15:**
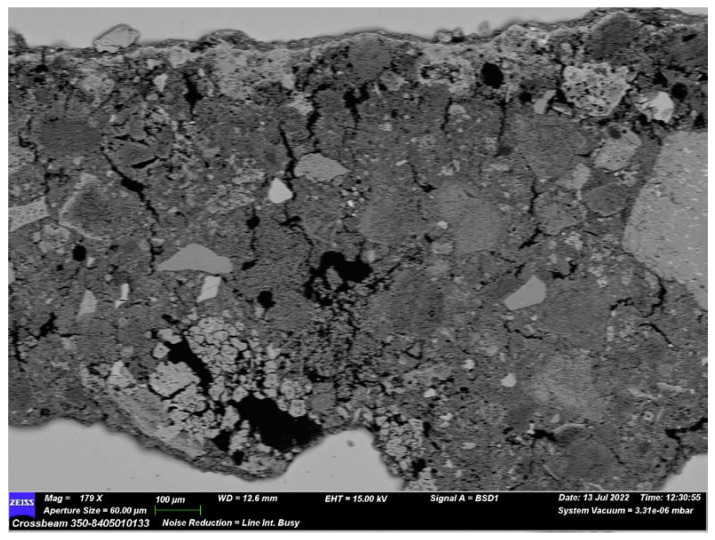
Image of sample ST1 obtained by SEM-EDS analysis.

By SEM observations, it is possible to distinguish three different layers in the ST1 sample (Figure 15):

- The bottom one corresponds to the first plaster layer characterized by moderately sorted aggregate, previously described by POM observations;
- The second one is a thin layer composed by the binder only;
- The upper one represents the pictorial film.

The presence of a thin binder layer can be explained as a tentative to smooth and prepare the surface for the pictorial film.

To compare the data from PXRF analysis, a chemical investigation of the pictorial layer was carried out. The compositional microanalysis using EDS performed punctually on the pictorial red film has detected a high level of iron attributable to the presence of iron oxides, confirming the PXRF data.

**Figure 16:**
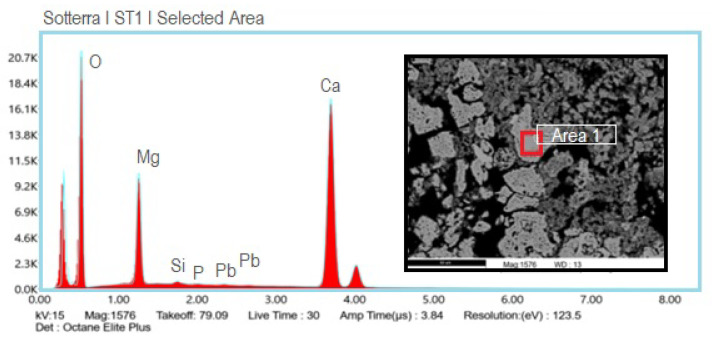
EDS spectra of the lump observed in the ST1 sample and related SEM image (to the right of the spectrum), highlighted in the red box as “Area 1”.

SEM analysis also provides important information about the binder used in the plaster. In this occasion, both the first layer and middle layer’s binder chemical compositions were investigated. Multiple punctual analyses on the lump, found by POM observation, were conducted for the characterization of the first layer’s binder. The result show both the calcium and magnesium peaks (Figure 16), which induces the hypothesis that the binder was obtained by the calcination of a dolomitic limestone.

The binder composition of the second layer shows a slight difference in comparison to the first binder layer. In fact, even if the magnesium peak is visible, the analysis records a lower magnesium contribution. This result can be explained considering the high humidity parameters that bring the magnesium in solution into the wall.

## 4. Discussion

The results obtained allowed the obtaining of important information about both the execution techniques and the conservation state of the wall painting investigated. As evidenced by ranking light observation, the painting was made by several *giornate.* The analysis of the overlapping correlations has helped to determine the executive sequence. The painting was realized following a preparatory drawing made with yellow ochre and earths, not observable under infrared lights.

The diagnostic investigations have clarified the painting stratigraphy. Two layers were detected: the first one is thicker, and it is composed of binder and aggregates; the second one consists of binder only, most likely applied to prepare the surface to the pictorial film. The aggregate of the first mortar layer is characterized almost entirely by carbonate rock fragments, while quartz crystals, biotite, quartzite grains, and oxides are more rarely found in all the samples observed. As the SEM-EDS analysis suggests, the binder was obtained by the calcination of a dolomitic lime. The differences in the binder composition between the first and the second mortar layer are explained considering the dissolution phenomena that bring the magnesium ions in solution into the porosity of the underlying material. This hypothesis is confirmed by the determination of moderate concentrations of magnesium ions in all of the investigated samples provided by ion chromatography. In fact, the dolomitic compound reacts with sulfate ions into the wall as follows: [14]

CaMg(CO_3_)_2_ + SO_4_^=^ → CaCO_3_ + MgSO_4_ + CO_3_^=^

The mineral-petrographic observation of thin and stratigraphic sections by POM not only provided evidence about plaster aggregate, but also allowed the revealing of the presence of degradation phenomena at the microscale.

Thanks to PXRF analysis, the chemical composition of the main pigment was detected. The main colors observed were analyzed preliminarily using a noninvasive technique, PXRF, to define the chromophores. For the red and yellow layers, ochres and earth-based pigments were used, as demonstrated by the signals of iron, manganese, silicon, aluminum, and potassium in all of the detected areas. A calcium peak, found in all investigated samples, is mostly attributable to the original plaster as well as the constitutive pigments. The pink pigment, used to paint the background, the Apostles’ robes, and the skin tones, considering that there are no significant elementary changes in pigment composition between the red layer and the pink one, is most likely obtained by mixing the red pigment with some lime or calcium carbonate-based pigment. The pictorial layer composition, consisting of iron oxides, was confirmed by SEM-EDS investigations as evidenced by iron peaks obtained by the spectra; the pigments found are typical of the period assigned to the painting [15].

The diagnostic campaign was also aimed at acquiring comprehensive knowledge of the alteration and degradation forms. The peculiar microclimate conditions, characterized by high relative humidity and constant temperature, represent the optimum for biological species growth [16,17]. The unstable thermohygrometric equilibrium may also be altered by external factors, connected to the heavy flows of visitors, which contribute to increasing the level of risk of biodeterioration phenomena. The biological growth observed on the painting surfaces was investigated by UV imaging. The red fluorescence emission related to the biological activity revealed the exact extension of the biofilm on the surface.

Moreover, UV imaging has helped to emphasize the recrystallization phenomena in the holes in the semidome. This is due to the reaction between the carbonate substrate and the gases naturally contained in the atmosphere. If an increase in the carbon dioxide concentration occurs, (caused, for example, by a conspicuous number of visitors,) the gas dissolves in condensed water, leading to the formation of carbonic acid. The product of the reaction between the above-mentioned acid and the calcium carbonate inside the original plaster will be calcium bicarbonate, a more soluble salt. The chemical process takes place as follows:

CO_2_ + H_2_O + CaCO_3_ → Ca(HCO_3_)_2_

On the contrary, when the thermohygrometric conditions vary and the evaporation of the aqueous solution occurs, the defined chemical equilibrium will shift towards the precipitation of calcium carbonate. The direct consequences are decohesion and abrasion of the pictorial film and the plaster due to repeated cycles of dissolution and crystallization.

The ions dispersed in the wall were detected by IC. In addition to calcium and sulfate concentrations, nitrates and chlorides are detected in low concentrations in the samples. The results interpretation suggests that the most abundant is the gypsum phase, which is stable even in extremely humid conditions. Most likely, calcium sulfate comes from the soil surrounding the building that for capillary action may crystallize on the wall surfaces.

The results obtained by IC and those obtained from the microclimatic monitoring can determine the crystallization risk. In fact, the microclimate data allow important indications for understanding the processes of precipitation of saline phases that take place in the substrate and to evaluate the seasonal crystallization phenomena. The precipitation mechanism is governed not only by salt-intrinsic properties such as its solubility, hygroscopicity, and deliquescence, but also by microenvironmental factors such as RH and its oscillations [18]. Crystallization might occur if the ambient relative humidity becomes lower than the equilibrium relative humidity (RH_eq_) of the saturated solution of the salt in the system. The calcium sulfate high-deliquescence RH (higher than 99% at 20 °C) indicates that the salt can crystallize even in extreme humid conditions. The observations conducted in situ and the collected data from continuous microclimatic analysis can be fundamental to determining the salt crystallization process.

In correspondence to the increase of external temperatures, during summer, inside the church a phenomenon of capillary rise is observed, and it is possible to determine the rising dump on the walls of the apse. For three months of the year, from June to September, the wall painting is interested in permanent saturation conditions. Referring to the parameters acquired by the data logger, it is evident that when the relative humidity values tend to decrease and drop below 100%, starting from the second half of September, crystallization processes occur. The efflorescence phenomenon is particularly evident from autumn to spring, in the area of the apse and on the surfaces of the adjacent wall paintings. On the contrary, starting from June, relative humidity values increase by up to 100%, causing the salt deliquescence [19].

The cycles of salt crystallization/dissolution and the wet/dry cycles happen annually, leading to deterioration phenomena which must be given due consideration.

Another conservation risk is represented by condensation on the surface of the painting. The condensation phenomenon is evident during summer, as the relative humidity increases up to 100% by the end of May. It can be stated that the process of condensation on surfaces contributes directly, following repeated cycles, to the dissolution of the carbonate matrix constituting the mural painting. The relative humidity values close to saturation, combined with significant carbon dioxide concentrations, favor the reaction between the condensed water, visible on the surface in the form of droplets, and CO_2_, leading to the formation of carbonic acid and ultimately causing the dissolution of the calcium carbonate of the original material.

Another interesting result discovered is that the fragile environmental equilibrium could be broken by external factors such as visitor flows inside the building. In fact, consequently to an intense visitor flow on May 15, the parameters of temperature show a significant increase of 2 degrees within 15 min. At the same time, the humidity relative values show an increase from 97% to 100%, most likely caused by the respiration of visitors that produced a dust layer on the wall surfaces [20].

The continued fluctuations of the thermohygrometric parameters must be taken into account, considering that they could cause the acceleration of the degradation processes.

## 5. Conclusions

A multianalytical approach was carried out to obtain an accurate comprehension of the mural painting in the apse of the Sotterra church. The results obtained from the diagnostic campaign were fundamental to defining the most appropriate conservation procedures to be adopted. Following this purpose, both in-field methods and laboratory-based analysis were conducted. In addition, the diagnostic campaign was concerned with the environmental characterization of the site provided by the microclimatic monitoring with detection of temperature (T) and relative humidity (RH).

The results obtained from nondestructive investigations by UV imaging and IR imaging were fundamental to achieving a preliminary knowledge of both original materials and degradation forms affecting the wall painting. PXRF analysis contributed to the characterization of the main pigments implied in the painting execution. For red, pink, and yellow shadows, natural mineral pigments such as ochres and earths were used.

Furthermore, POM observations allowed the determining of the painting stratigraphy, provided information about mortar aggregate, and characterized at the microscale the conservation state of the plaster. The mural painting consists of two plaster layers characterized by lime-based binder, as determined by SEM-EDS investigation.

As evidenced by the IC results, the most abundant saline phase is represented by calcium sulfate. The analysis of microclimatic data, combined with the diagnostic results obtained, allow the establishment of the crystallization and the condensation risks which occur yearly on the painting surfaces. Finally, access to the church should be controlled and tourist visits must be limited in order to preserve the paintings.

## Figures and Tables

**Table 1:** Measurement areas of the apse wall painting.

Area	Measurement Area	Type
P1	Lower decorative frame	Yellow layer
P2	Robe of the Apostle	Red layer
P3	Robe of the Apostle	Red layer
P4	Background	Pink layer

**Table 2:** Samples taken from the apse mural painting.

Samples	Sampling Area	Employed Techniques
ST1	Lower decorative frame	POM, SEM-EDS
ST2	Preparatory layer	IC
ST3	Preparatory layer	IC
ST4	Background	IC
ST5	Background	IC
ST6	Background	IC
ST7	Lower decorative frame	POM, IC
ST8	Lower decorative frame	POM, IC

**Table 3:** PXRF elemental composition of the apse mural painting. Minor elements are indicated in parentheses.

Area	Type	Chemical Elements by PXRF	Identified Chromophore
P1	Yellow layer	Ca, Fe, Si, K, Al, Mn (S, Ti, Sr)	Yellow ochres
P2	Red layer	Ca, Fe, Si, K, Al, Mn, Ni (S, Ti, Sr)	Red ochres/earths
P3	Red layer	Ca, Fe, Si, K, Al, Mn (S, Ti, Sr)	Red ochres/earths
P4	Red layer	Ca, Fe, Si, K, Al, Mn (S, Ti, Sr)	Red ochres/earths

## Data Availability

Not applicable.
